# Bifunctional rare metal-free electrocatalysts synthesized entirely from biomass resources

**DOI:** 10.1080/14686996.2021.2020597

**Published:** 2022-01-18

**Authors:** Hiroshi Yabu, Kosuke Ishibashi, Manjit Singh Grewal, Yasutaka Matsuo, Naoki Shoji, Koju Ito

**Affiliations:** a Advanced Institute for Materials Research (WPI-AIMR), Tohoku University, Sendai, Japan; b Institute of Multidisciplinary Research for Advanced Materials (IMRAM), Tohoku University, Sendai, Japan; c Headquarter, AZUL Energy, Inc., Sendai, Japan; d Institute for Electronic Science (RIES), Hokkaido University, Japan; e Center for the Cooperation of Community Development and Research Promotion, Miyagi University, Miyagi, Japan

**Keywords:** Biomass resources, electrocatalysts, oxygen reduction, oxygen evolution, cellulose nanofibers, 50 Energy Materials, 103 Composites < 100 Materials

## Abstract

The oxygen reduction reaction (ORR) and oxygen evolution reaction (OER) are important processes for various energy devices, including polymer electrolyte fuel cells, rechargeable metal–air batteries, and water electrolyzers. We herein report the preparation of a rare metal-free and highly efficient ORR/OER electrocatalyst by calcination of a mixture of blood meal and ascidian-derived cellulose nanofibers. The obtained carbon alloys showed high ORR/OER performances and proved to be promising electrocatalysts. The carbon alloys synthesized entirely from biomass resources not only lead to a new electrocatalyst fabrication process but also contribute to CO_2_ reduction and the realization of a good life-cycle assessment value in fabrication of a sustainable energy device.

## GRAPHICAL ABSTRACT

**Figure:**
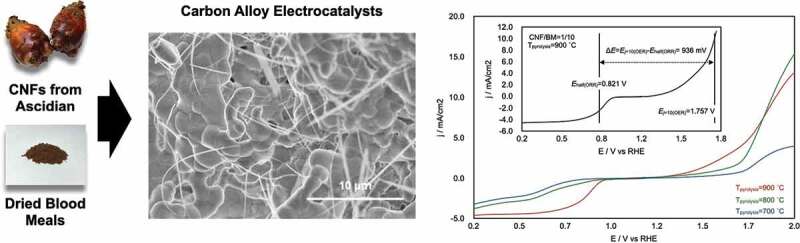

## 1. Introduction

The oxygen reduction reaction (ORR) and oxygen evolution reaction (OER) are important processes for various energy devices, including polymer electrolyte fuel cells (PEFCs), rechargeable metal–air batteries, and water electrolyzers. At the cathode of PEFCs [1] and metal–air batteries [2] [3,4],, O_2_ molecules in the air are reduced to hydroxyl ions via the ORR and efficient catalysts are needed to lower the overpotential of the reaction. To achieve highly efficient energy conversion, Pt supported on carbon black (Pt/C) has been used as an ORR catalyst. However, the use of Pt, which is expensive and resource-constrained, has hindered the widespread use of these batteries [5]. To avoid resource constraints and reduce costs, catalytic materials that do not use heavy or precious metals such as Pt are needed [6]. The OER occurring at the cathode of rechargeable metal–air batteries and at the anode of water electrolysis systems [7] is also an important process for efficient O_2_ generation in such devices [8]. Pt, Ir, Ru, and other precious-metal catalysts have been used to reduce the OER overpotential, which is associated with the bottleneck process in water electrolysis.

For decades, bifunctional catalysts have been extensively explored as alternatives to precious-metal catalysts [9], and numerous materials, including core–shell metal nanoparticles [10] and metal oxide nanoparticles [11], have been substituted for rare metals and rare-earth metals in ORR and OER catalysts. In addition, the literature includes reports that show carbon alloys (CAs) containing pyridine groups and metal atoms M surrounded by four pyridines (MN_4_ structure, especially FeN_4_) embedded in a graphite structure exhibit substantially enhanced ORR activity [12]. Consequently, the development of CAs containing pyridine groups and MN_4_ structures has been intensively investigated [13] [14–16]. Ozaki and coworkers reported the preparation of CAs containing FeN_4_ structures by calcination of iron phthalocyanines and a polymer resin [17]. Molecular adsorption of iron phthalocyanine and other derivatives to form carbon composites with FeN_4_ structures has also been examined [18] [19,20]. However, these carbon sources are usually synthetic and require multistep synthesis routes involving substantial CO_2_ emissions. Thus, these materials have a high environmental impact in terms of life-cycle assessment (LCA) given that the number of PEFCs, metal–air batteries, and electrolyzers will increase even though they will incorporate conventional alternative electrocatalysts.

Sustainable resources, including recycled materials and biomass resources, provide a promising path to reducing carbon footprints associated with the synthesis of electrocatalysts. In particular, the synthesis of heteroatom-doped electrocatalysts from biomass resources is a substantial challenge toward realizing a sustainable energy source [21] [22]. The literature includes reports of the synthesis of electrocatalysts from biomass resources [23] [24,25]. Hemoglobin and blood meal (BM), which is a dried waste blood from the meat processing of livestock, contain large amounts of heme protein and have strong potential as an FeN_4_ resource for electrocatalysts [26]. Huge amounts of BMs (over thousands of tons per year) were produced and most of them were thrown away and causes the environmental pollution since it increases biochemical oxygen demand (BOD) of rivers, lakes and other water environments. Only a part of them were used as low-cost fertilizers containing high amount of Fe, N and P resources. The synthesis of ORR catalysts via pyrolysis of pig blood has been reported [27] [28]. Normally, to achieve sufficient catalytic activity and electrical conductivity to enable the pyrolysis of pig blood or BM itself, metal/metal oxide sources [29] [30], and carbon materials [31] [32–34], are added to the precursors. However, the literature contains few reports on the synthesis of electrocatalysts from other biomass resources.

Cellulose nanofibers (CNFs) are widely used in structural components and as reinforcements for polymer materials because of their high mechanical and thermal stabilities, which arise from the highly crystalline structure of cellulose. Furthermore, amount of ascidian tunicates as a biomass is also huge enough, which is over 7,000 tons/year obtained as same as BMs, which is enough amount to produce CNFs over tens tons/year. Comparing with wood biomass, the advantage of CNFs from ascidian tunicates is they can be harvested every year. Interestingly, CNFs obtained from marine organisms, including seaweeds and ascidians, exhibit greater crystallinity [35] and a larger crystalline size than wood-based CNFs. In addition, calcining marine CNFs leads to the formation of carbon materials with highly graphitic structures, which in turn results in highly conductive carbons [36] [37]. Given the high conductivity of pyrolyzed CNFs prepared from marine bioresources, we anticipated that combining BMs and CNFs from marine bioresources would be a promising approach for preparing ORR/OER electrocatalysts with high catalytic activity and high electron conductivity. Actually, we have recently reported pyrolysis of polydopamine-coated cellulose nanocrystals obtained from ascidians show good ORR activities [38].

In the present paper, we report the synthesis of bifunctional heteroatom-doped CA electrocatalysts for both the ORR and the OER by pyrolyzing BM and CNFs from ascidians. The chemical properties and electrochemical performance of the synthesized CAs are discussed.

## 2. Experimental details

### 2.1. Materials

BM from pig was kindly supplied by Tomikura Sangyo. An alkaline aqueous dispersion of CNFs was obtained from ascidians in the Miyagi prefecture, Japan, using a previously reported sodium hypochlorite treatment method [39]. Details of the extraction process of CNFs from tunicates of ascidian are provided in the supporting information, S1. To remove excess sodium hypochlorite, the CNFs were washed three times with membrane-filtered water and collected by centrifugation (~3000 rpm). After purification, a 2 wt% dispersion of CNFs in membrane-filtered water was prepared.

### 2.2. Synthesis of carbon alloys

The BM and CNFs were mixed in various weight ratios by hand grinding in water, and the resultant mixtures were dried at room temperature *in vacuo*. In order to avoid those heterogeneity, we mixed and grinded to homogenized the samples before pyrolysis. The pyrolyzed samples were derived from the same crude materials after mixing and grinding, therefore we considered that there are no intended heterogeneities. The dried samples were pyrolyzed in an electric oven at 700, 800, or 900°C *in vacuo* for 3 h. The heating rate was 20°C/min. After pyrolysis, the sample was slowly cooled to room temperature. After this pyrolysis process, powder-like CAs were obtained.

### 2.3. Characterization

Structures of pyrolyzed samples were observed using a scanning electron microscope (S-5200, Hitachi, Japan) and a transmission electron microscope (H-7650, Hitachi, Japan). *In situ* energy-dispersive X-ray analysis (EDX) spectra of samples were obtained using a transmission electron microscope equipped with an EDX system (EDAX, Genesis) and operated at an acceleration voltage of 100 kV. X-ray photoelectron spectroscopy (XPS) measurements were performed with a JPS9200 spectrometer (JEOL; Al Kα, 10 kV, 10 mA). A wide scan was performed from 0 to 1400 eV in 1 eV steps, and a narrow scan was performed for each element in 0.1 eV steps. Thermogravimetric analysis was performed on RIGAKU Thermo plus EvoII TG-DTA8210 at a heating rate of 10°C/min under a nitrogen atmosphere.

### 2.4. ORR/OER measurements

The ORR/OER performances were evaluated by linear-sweep voltammetry (LSV) measurements using a potentiostat (2325, BAS, Japan). Catalyst inks for each sample were prepared by dispersing 0.82 mg of catalyst in a 1 mL solution consisting of 6 μL Nafion (527,084, Sigma-Aldrich, USA), 334 μL isopropyl alcohol, and 84 μL water via sonication for 5 min. Twenty microliters of the ink was then cast onto a glassy carbon (GC, BAS, Japan) insert of a rotating ring-disk electrode (RRDE; 4 mm diameter, BAS, Japan) and dried. The catalyst loading on the electrode was 300 μg/cm^2^. A Pt wire and a Ag/AgCl electrode were inserted into the electrolyte as reference and counter electrodes, respectively. A 0.1 M KOH solution bubbled with N_2_ or O_2_ for 30 min was used as the electrolyte. In addition, 3 M methanol was added to an O_2_-saturated 0.1 M KOH solution for evaluation of the methanol oxidation activity of the catalysts.

The potential vs Ag/AgCl was converted to the reversible hydrogen electrode (RHE) scale using the following equation:

*E*(*vs RHE*) *= E*(*vs Ag*/*AgCl*) *+* 0.197 *+ *0.059 *V*·pH (1)

The number of electrons (*n*) involved in the ORR was calculated according to the Kouteck*ý*–Levich (K–L) equation:

(2) $$\frac{1}{J}=\frac{1}{J_{k}}+\frac{1}{J_{d}}=\frac{1}{nFAkC_{O_{2}}}+\frac{1}{0.62nFAD_{O_{2}}^{2\;/\;3}\nu^{-1\;/\;6}C_{O_{2}}\omega^{1\;/\;2}}$$

where *J, J*_k_, and *J*_d_ are the measured, kinetic, and diffusion-limiting current, respectively; *F* is the Faraday constant (96,485 C/mol); *A* is the electrode area (0.1256 cm^2^); *k* is the rate constant for oxygen reduction (M/s); *D*_O2_ is the diffusion coefficient of O_2_ in the electrolyte (1.93 × 10^−5^ cm^2^/s); *ν* is the viscosity of the electrolyte solution (1.009 × 10^−5^ cm^2^/s); *C*_O2_ is the saturated concentration of O_2_ in the electrolyte (1.26 × 10^−6^ mol/cm); and *ω* is the angular rotation rate.

The number of electrons *n* involved in the ORR was also calculated using the RRDE results and the following equation:

(3) $$n=\frac{4I_{D}}{I_{D}+\frac{I_{R}}{N}}$$

where *I*_D_ and *I*_R_ are the current densities of the disk and ring electrodes, respectively, and *N* is the capture efficiency (0.42). Reproducibility of the performance for several times from different crude materials.

## 3. Results and discussion

### 3.1. Chemical characterization of CAs

**Figure 1.**
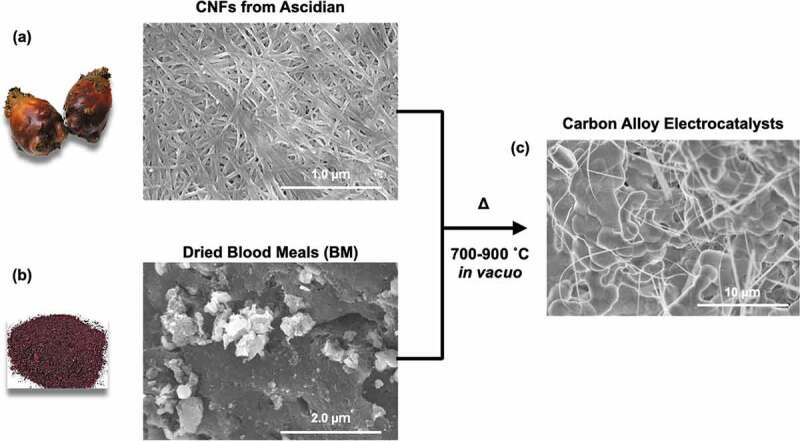
SEM images of the original CNFs (a) and BM (b) and the CAs (c).

CAs with various CNF/BM ratios were prepared by pyrolysis of the CNFs and dried BM at 700, 800, or 900°C *in vacuo*. Sample compositions and pyrolyzed temperature (*T*_pyrolysis_) are listed up in Table 1. Figure 1 shows typical scanning electron microscope (SEM) images of CNFs extracted from ascidians and dried BM. From the SEM image, the average width and length of the CNFs was 28.9 nm (see supporting information, S1) and greater than several micrometers, respectively. TGA curves and SEM images of pyrolyzed samples were shown in the supporting information, S7 and S8. From TGA curves, the weight of CNF almost burned at 900°C, but it still existed small percentage (1 ~ 2%). I agree with the reviewer’s comment that main component of carbon alloys derived from BMs. From those TGA curves, only a small amount of CNF derived carbons should exist in the carbon alloys.

**Table 1.** List of samples and their representative electrocatalytic performances measured at pH 13

Sample names	*C_CNFs_* [%]	*C_BM_* [%]	*T_pyrolysis_* [°C]	*E_on-set/ORR_* [V vs RHE]	*E_half/ORR_* [V vs RHE]	*I_max/ORR_* [mA/cm^2^]	*N*	*E_on-set/OER_* [V vs RHE]	*η* [mV]
CA-1/2-700 °C	33.3	66.7	700	0.853	0.604	2.11	2.11	1.698	468
CA-1/2-800 °C	33.3	66.7	800	0.937	0.597	1.93	2.92	1.689	459
CA-1/2-900 °C	33.3	66.7	900	0.988	0.829	2.41	3.19	1.397	167
CA-1/5-700 °C	16.7	83.3	700	0.886	0.606	4.47	2.18	1.682	452
CA-1/5-800 °C	16.7	83.3	800	0.938	0.655	1.80	3.31	1.688	458
CA-1/5-900 °C	16.7	83.3	900	0.988	0.839	3.93	3.69	1.347	117
CA-1/10-700 °C	9	91	700	0.870	0.616	4.47	2.41	1.682	452
CA-1/10-800 °C	9	91	800	0.945	0.657	1.98	3.93	1.673	443
CA-1/10-900 °C	9	91	900	1.011	0.821	4.84	3.95	1.393	163
Pt/C	-	-	-	1.035	0.850	7.29	4.1	1.400	170

BM is a mixture of blood proteins and lipids; its SEM images therefore show no clear characteristic structures. Figure 1(c) shows a typical SEM image of a pyrolyzed mixture of CNFs and BM. After pyrolysis, the CNFs and BM were transformed into a thin fibrous network and bulk sintered bodies, respectively.

**Figure 2.**
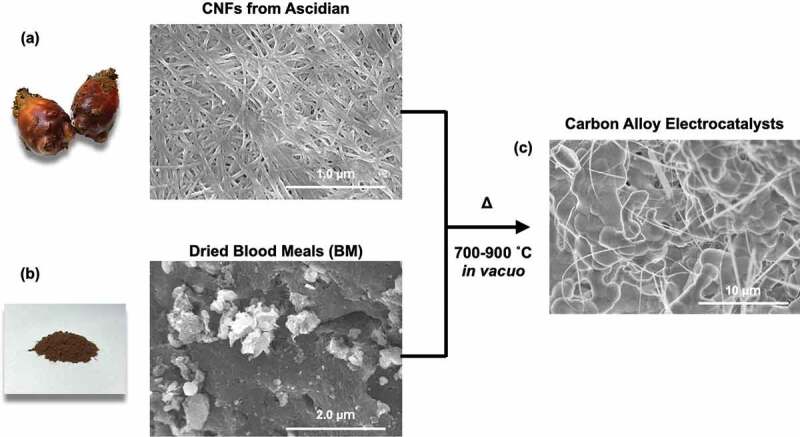
A typical EDX spectrum of a CA (BM/CNF = 10/1, pyrolyzed at 900°C) (a) and the Fe/C (b) and P/C (c) ratios as functions of *T*_pyrolysis._

To analyze the chemical compositions of the CAs prepared by pyrolysis of the BM and CNF mixtures, TEM–EDX and XPS analyses were performed. Figure 2(a) shows a typical TEM–EDX spectrum of one of the CA samples. Peaks attributable to C and O of the pyrolyzed CNFs and peaks attributable to the P and Fe of BM are clearly observed. The Ca was a contaminant from seashells, which were used as scaffolds for the ascidians during aquaculture. The Cu and other small peaks were originated from the Cu grids and the sample holder, respectively. Because heteroatom-doped carbons exhibit drastically enhanced electrocatalytic activity, the incorporation of N, S, P, and metals into carbon materials is important for understanding the catalytic properties of these materials. The TEM–EDX spectra show clear peaks for P and Fe, which are known as dopants for electrocatalysts. Figure 2(b) and 2(c) show Fe/C and P/C ratios of each sample in terms of pyrolysis temperature (*T*_pyrolysis_). From this result, Fe and P atoms are well incorporated into the resultant carbons pyrolyzed at 900°C. Nitrogen concentrations in samples are relatively lower than other elements in the XPS results, therefore, the low sensitivity of EDX spectroscopy for light elements and low abundance of nitrogen comparing to carbon may have prevented it from being observed.

**Figure 3.**
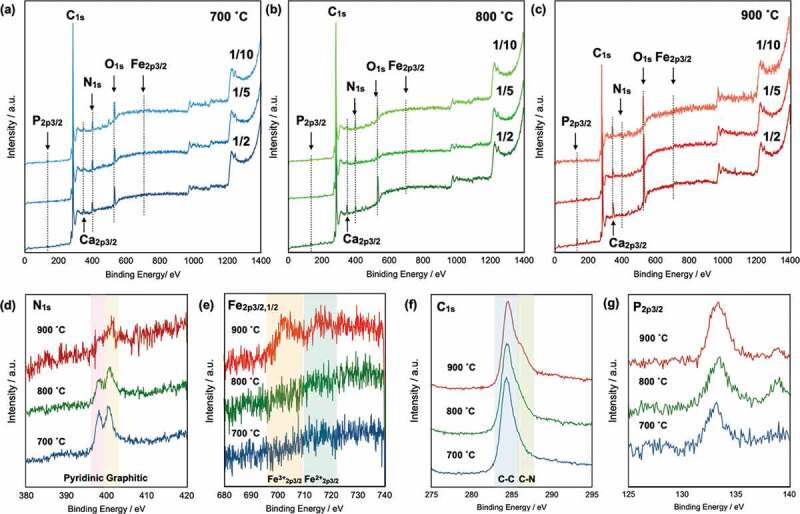
Wide-scan XPS spectra of CAs pyrolyzed at 700°C (a), 800°C (b), and 900°C (c); narrow-scan XPS spectra of CAs prepared from precursors with a CNF/BM ratio of 1/2: N_1*s*_ (d), Fe_2*p*_ (e), C_1*s*_ (f), and P_2*p*3/2_ (g).

The wide-scan XPS spectra show that the obtained CAs contain N, O, and P atoms (Figure 3(a)–(c)). The strong C_1*s*_ peak at 280–290 eV indicates that the pyrolyzed materials were converted into carbon materials. Small peaks attributed to P_2*p*3/2_, N_1*s*_, and O_1*s*_ were observed at 133, 401, and 531 eV, respectively. In addition, small peaks attributed to Ca_2*p*3/2_ were observed at 350 eV, which is a contaminant from the original CNF.

To discuss the effect of the *T*_pyrolysis_ on the carbon structures, narrow-scan XPS spectra were recorded for pyrolyzed samples. Figure 3(d) shows the narrow-scan spectra of the CA-1/2 samples pyrolyzed at 700°C (blue solid line), 800°C (green solid line), and 900°C (red solid line). Two distinct peaks attributable to pyridinic N and graphitic N were observed. Proteins do not normally contain pyridinic structures; the pyridinic N is therefore attributed to heme structures in the blood cells. Both peaks gradually decreased in intensity with increasing *T*_pyrolysis_ because the N-containing compounds are easily decomposed into N_2_ and NO*_x_* gases at elevated temperatures. However, small peaks remained in the spectrum of the CA pyrolyzed at 900°C. In addition, Fe peaks were not observed in the spectra of the CAs pyrolyzed at 700 and 800°C, whereas small Fe^3+^_2*p*3/2_ and Fe^2+^_2*p*3/2_ peaks emerged in the spectrum of the CA pyrolyzed at 900°C (Figure 3(e)). These results indicate that Fe atoms were incorporated into the CA in the case of pyrolysis at 900°C. These results are consistent with the EDX analysis results, which indicate that the Fe/C ratio of the sample prepared at *T*_pyrolysis_ = 900°C is substantially greater than the Fe/C ratios of the samples prepared at *T*_pyrolysis_ = 700°C and 800°C. In the narrow-scan XPS spectra of C_1*s*_ (Figure 3(f)), peaks attributable to C–C bonds are clearly observed; however, shoulder peaks attributable to C–N bonds increase in intensity with increasing pyrolysis temperature [40]. These simultaneous increases in C–N and Fe peak intensities indicate the formation of FeN_4_ structures based on the complex of Fe and pyridinic nitrogen in carbon. Because the FeN_4_ structure is important for the ORR, the CAs are expected to exhibit high ORR performance. In addition, the narrow-scan XPS spectra show that phosphate, which is a component of the phospholipids in the BM, was also incorporated into the CAs irrespective of the *T*_pyrolysis_ (Figure 3(g)). The peak intensity and sharpness gradually increased with increasing *T*_pyrolysis_.

These chemical characterization results indicate that the CNF/BM mixtures were decomposed into CAs. Moreover, they show that heteroatoms such as N and P were incorporated into the CAs pyrolyzed at 700 or 800°C and that N, P, and Fe were incorporated into the samples pyrolyzed at 900°C. The amount of Fe in 700 and 800 0 C is less and thus Fe-N_4_ structures were less formed. The P and Fe contents increased with increasing BM content. The content of heteroatoms was changed with changing the pyrolysis temperature. Especially, same metal complex introduction was observed in the literature [41]. Metal complexes were successfully introduced to carbon alloys from metal ion and carbon precursors at high pyrolysis temperature due to high chemical reactivity and the content of metal complexes increased with increasing the pyrolysis temperature.

### 3.2. ORR performance

**Figure 4.**
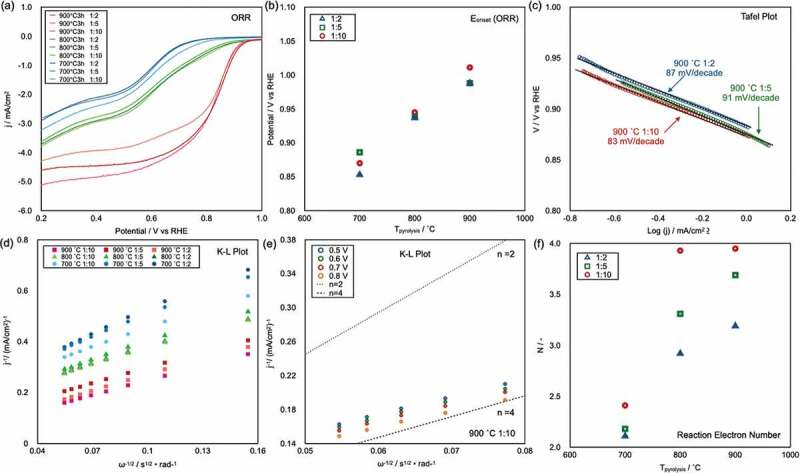
(a) Linear-sweep voltammograms of CAs recorded at an RRDE rotation speed of 1600 rpm in pH 13 KOH (aq). (b) Plot of *E*_onset_ as a function of the pyrolysis temperature (*T*_pyrolysis_). (c), (d) Tafel plots of CAs pyrolyzed at 900°C and K–L plots of the corresponding samples and CAs obtained from CNF/BM = 1/10 pyrolyzed at 900°C. (f) Electron transfer number *N* as a function of *T*_pyrolysis._

Figure 4(a) shows the linear-sweep voltammograms of all of the prepared CAs, as recorded at 1600 rpm in 0.1 M KOH(aq) (pH 13) in the voltage range from 0.2 to 1.0 V vs RHE (see supporting information, S3, which shows LSV curves recorded at different rotation speeds for each sample). Parameters used to characterize the ORR performance of electrocatalysts include the onset potential (*E*_onset_), half-wave potential (*E*_half_), and maximum current density (*I*_max_). High *E*_onset_, *E*_half_, and *I*_max_ values indicate high ORR catalytic performance. The curves clearly show that the CA samples pyrolyzed at 900°C (red solid lines) exhibit better ORR performance than the CAs pyrolyzed at 800°C (green solid lines) and 700°C (blue solid lines). The sample names and measured *E*_onset_, *E*_half_, and *I*_max_ values are shown in Table 1. The *E*_onset_ of all samples are plotted as a function of *T*_pyrolysis_ in Figure 4(b). The *E*_onset_ value linearly increased with increasing *T*_pyrolysis_. The best ORR performance was observed in the case of CA-1/10-900°C; its *E*_onset_, *E*_half_, and *I*_max_ were 1.01 V, 0.821 V, and 4.84 mA/cm^2^, respectively. Notably, these values are similar to those reported for Pt/C [9].

The rate-limiting process of the ORR on catalysts can be estimated from Tafel plots. The Tafel plots for the ORR on the CAs pyrolyzed at 900°C are shown in Figure 4(c). The Tafel slopes of the respective catalysts range from 82 to 91 mV/decade, indicating that the rate-limiting step of the ORR on these catalysts was the adsorption of O_2_ molecules onto active sites, which is the same rate-limiting step of the ORR on Pt/C [42]. Two pathways exist for the ORR: a two-electron (2e^−^) process, in which O_2_ molecules are reduced to H_2_O_2_, and a four-electron (4e^−^) process, which offers complete reduction from O_2_ molecules to OH^−^. To maximize the battery performance, an electrocatalyst should catalyze the ORR *via* 4e^−^ process.

Figure 4(d) shows the K–L plots calculated via equation (2) using data from the linear-sweep voltammograms recorded under various RRDE rotation speeds (one method for evaluating the reaction electron number (*N*) of the ORR) for whole samples at 0.6 V, and Figure 4(e) shows the plot for the CAs prepared from CA-1/10-900°C at different potentials. The linearity of the K–L plots shows the reaction kinetics for dissolved O_2_ and the electron transfer numbers of the ORR. In the K–L plots of whole samples, linear slopes are observed; the slopes gradually decrease with increasing *T*_pyrolysis_. This result indicates that the *N* of the ORR increased with increasing *T*_pyrolysis_. From Figure 4(e), the K–L plots of CAs prepared from CA-1/10-900°C were constantly found to be close to the *N* = 4 line, which indicates that the ideal 4e^−^ process was achieved on this catalyst. The *N* value was also evaluated using equation (2) on the basis of HO^−^ yields measured using an RRDE in 0.1 M KOH. Figure 4(f) shows a plot of *N* as a function of *T*_pyrolysis_. The *N* values increase with increasing *T*_pyrolysis_, similar to the K–L plot data.

The EDX and XPS chemical analysis results discussed in the previous section indicate that pyridinic N, P and FeN_4_ structures were formed in the case of CAs pyrolyzed at 900°C. Normally, pyridinic N and FeN_4_ structures strongly enhances the performance of ORR. In order to reveal the catalytic center of ORR, the disc electrode was immersed in 10 mM KCN solution to block Fe atom with CN. By this poisoning experiment, ORR performance decrease when the FeN_4_ structure is the catalytic center. From the LSV curves before and after poisoning experiment, the *E*_onset_ value slightly decreased (See supporting information, S2). Given that the pyridinic N and FeN_4_ structures both enhance the ORR performance, especially pyridinic N is the catalytic center. The N1s peak decreased with increasing pyrolysis temperature but ORR performance increased. This was caused by successful formation of Fe-N_4_ structures even though the content of N was decreased. Off cause, N-doped graphene also increase the ORR catalytic activities, but Fe-N_4_ structure is more sufficient for ORR catalytic activities. Therefore, ORR activities increased with increasing the pyrolysis temperature.

The linear-sweep voltammograms of the GC, BM, and CNFs pyrolyzed at 900°C were recorded using an RRDE, like the voltammograms of the other CA samples (see supporting information, S4). A comparison of the results shows that the BM-900°C and CNF-900°C exhibited better ORR performance than the bare GC electrode; however, their performance was relatively lower than that of CA-1/10-900°C. Notably, the alloyed materials exhibited the best ORR performance and the pyrolyzed original materials exhibited worse performance. ORR performance of carbon alloys from those composites showed superior performance than carbons obtained from BM or CNF, respectively as shown in the supporting information, S4. And also, the carbons pyrolyzed only from CNFs showed higher ORR performance than that of BMs. Therefore, carbon alloys from BMs had inferior performance than those two carbons and it can not be explained if only BMs contribute to the performance. These results suggest that the alloying process enhances the ORR performance.

### 3.3. OER performance

**Figure 5.**
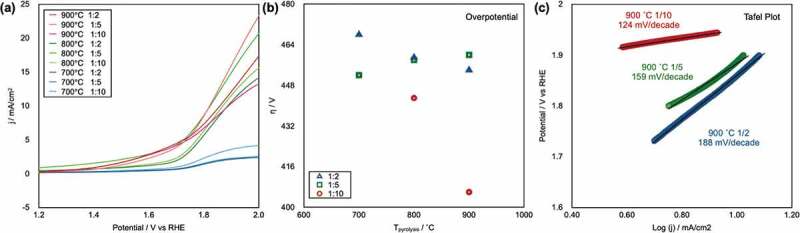
(a) Linear sweep voltammograms of CAs, as recorded at a rotation speed of 1600 rpm in pH 13 KOH (aq). (b) Plots of the overpotential (*η*) as a function of the *T*_pyrolysis_. (c) Tafel plots of the OER on CAs pyrolyzed at 900°C.

Linear sweep voltammograms for the OER of all the CAs are shown in Figure 5(a). The CAs pyrolyzed at 700°C exhibit a high *E*_onset_; however, the CAs pyrolyzed at 800°C and 900°C show lower *E*_onset_ values (Table 1). The OER overpotential (*η*) values obtained from the *E*_onset_ results are plotted as a function of *T*_pyrolysis_ in Figure 5(b). This plot shows that the *η* of the CAs was not affected by the *T*_pyrolysis_, whereas the *η* of the CA-1/10 series decreased with increasing *T*_pyrolysis_; it also shows that the OER performance of the CA-1/10-900°C was prominent. The *E*_onset(OER)_ hierarchy was IrO_2_/C ≈ CA-1/100–900°C > Pt/C ≫ GC (see supporting information, S4). The Tafel slopes of the CA-1/2-900°C, CA-1/5-900°C, and CA-1/10-900°C specimens were 188, 159, and 124 mV/decade, respectively (Figure 5(c)). A smaller Tafel slope normally indicates a smooth reaction during the OER, and the results strongly indicate high performance of the CA-1/10-900°C sample.

According to the literature, CAs containing heteroatoms such as N and P show high OER performance [41]. Because the CA-1/10-900°C contains both N and P, and because the XPS signal of P in this CA was obviously sharp, these heteroatoms played important roles in the OER. From the XPS analysis, it is clear that P was incorporated in the carbon alloys. P should be incorporated into the graphite network therefore it is very difficult to characterize local chemical structures around P atoms, but from literatures, C-P and P-O peaks were found in the peak of narrow scan XPS spectra of P2p at 132.0 eV and 133.5 eV [43]. Those peaks were also found in our results, which indicates P atoms were incorporated in the graphite network and some of them were oxidized. In addition, the OER process requires high electron conductivity of the catalysts because a high voltage is applied to the electrodes; low conductivity would result in chemical degradation of the catalysts and the electrodes. This highly developed graphitic structure of carbon alloys originated from highly crystalline structures of ascidian CNFs also contributes to the high OER performance. From the Raman scattering spectra (See supporting information, S6), broad peaks attributed to defect (D) and graphite (G) structures were found at 1,350 cm^−1^ and 1580 cm^−1^, respectively. The ratio between those two peak intensities represents quality of the carbon materials. From the Raman spectra of CA-1/10-700, 800, and 900 °C, the D/G ratio is almost constant in each case, and the ratio was c. a. 1, which means the quality of the carbon obtained from CNFs and BMs were close to commercially available carbon blacks.

### 3.4. Total ORR/OER performance of CAs

**Figure 6.**
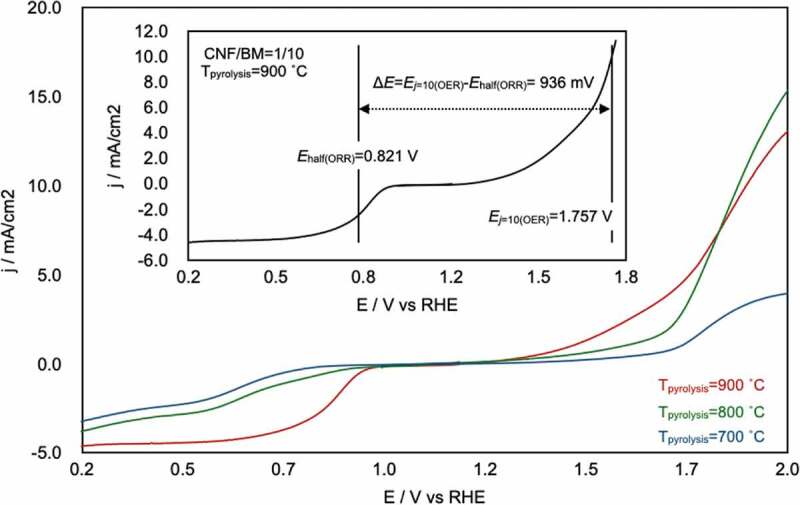
Overall linear-sweep voltammograms of CA-1/10-700°C, −800°C, and −900°C and calculation of Δ*E* (inset). From this plot, the potential at *j* = 10 mA/cm^2^ on the OER (*E_j_*_=10(OER)_) and the half-wave potential of the ORR (*E*_half(ORR)_) were determined to be 1.757 and 0.821 V, respectively. The difference between these values, Δ*E*, was 936 mV.

Figure 6 shows the overall linear-sweep voltammograms for the CA-1/10-700°C, −800°C, and −900°C, which provide information about both the ORR and OER properties of the CAs. These plots clearly show that *T*_pyrolysis_ = 900°C was the best pyrolysis temperature for realizing high-performance bifunctional electrocatalysts. The overall oxygen electrode activity can be evaluated by the variance of the OER and ORR metrics (Δ*E* = *E_j_*_=10(OER)_ − *E*_half(ORR)_) [44,45]. The smaller the Δ*E* value, the closer the electrode to an ideal reversible oxygen electrode. The Δ*E* value was calculated from total linear-sweep voltammograms of CA-1/10-900°C, and the value was estimated as 936 mV (inset of Figure 6). This value is similar to that of highly active electrocatalysts that include a noble metal (e.g. Pt/C [46], Δ*E* = 940 mV; Ir/C [47], Δ*E* = 920 mV; see supporting information S5), transition metal (e.g. CaMn_4_O*_x_* [47], Δ*E* = 1040 mV; Ni*_x_*O*_y_*/N-doped C [46], Δ*E* = 930 mV), or non-metal material (e.g. N-doped graphene/carbon nanotubes [48], Δ*E* ≈ 1000 mV).

It is well-known that Fe-N_4_ structures show high ORR catalytic property and it was generally understood by high affinity of Fe atom surrounded by four pyridinic nitrogen atoms to oxygen molecules [20]. P was assisted the adhesion of oxygen molecules in the ORR activities [43]. Regarding OER, edge nitrogen atom has high affinity to OH^−^ ions, this was also associate with the high performance of OER [20].

### 3.5. Conclusions

In the present paper, we presented high-performance ORR/OER bifunctional electrocatalysts prepared by pyrolysis of all-biomass resources (CNFs and BM mixtures). A high pyrolysis temperature enabled the formation of heteroatom doped CAs containing N, P, and Fe. The best electrocatalyst showed high ORR/OER performance comparable with that of previously reported noble- and transition-metal electrocatalysts and CAs such as N-doped graphene. Notably, we did not use any synthetic materials when preparing the high-performance bifunctional electrocatalysts. Both the methodology developed for preparing the electrocatalysts and the electrocatalysts themselves will contribute to realizing a sustainable society by reducing CO_2_ emissions associated with battery production and will contribute to the high LCA of energy devices such as PEFCs, metal–air batteries, and water electrolyzers.

## Supplementary Material

Supplemental Material
