# Supplementary material for: Bifunctional rare metal-free electrocatalysts synthesized entirely from biomass resources
Source: Sci Technol Adv Mater. 2022 Jan 18;23(1):31–40. doi: 10.1080/14686996.2021.2020597 (PMC8774140; doi:10.1080/14686996.2021.2020597)
Supplement: Supplemental Material [file TSTA_A_2020597_SM0434.docx]

**Supporting Information**

**Bifunctional rare metal-free electrocatalysts synthesized entirely from biomass resources**

Hiroshi Yabu^1,2,3,*^, Kosuke Ishibashi^1^, Manjit Singh Grewal^1^, Yasutaka Matsuo^4^, Naoki Shoji^5^ and Koju Ito^3^

^1^ WPI-Advanced Institute for Materials Research (AIMR), Tohoku University, 2-1-1, Katahira, Aoba-Ku, Sendai 980-8577, Japan

^2^ Institute of Multidisciplinary Research for Advanced Materials (IMRAM), Tohoku University, 2-1-1, Katahira, Aoba-Ku, Sendai 980-8577, Japan

^3^ AZUL Energy, Inc., 1-9-1, Ichibancho, Aoba-Ku, Sendai 980-0811, Japan

^4^ Institute for Electronic Research (RIES), Hokkaido University, N21W10, Sapporo 001-0021, Japan

^5^ Miyagi University, 1-1, Gakuen, Taiwa-Cho, Kurokawa-Gun, Miyagi 981-3298, Japan

**Appendix**

**Supporting Information, S1.** Extraction protocol of CNFs from ascidian. 2

**Supporting Information, S2.** LSV curves of a carbon alloy before/after CN^-^ poisoning. 3

**Supporting Information, S3.** LSV curves of carbon alloys with different rotating speed. 4

**Supporting Information, S4.** SEM images and LSV curves of pyrolyzed BMs and CNFs. 5

**Supporting Information, S5.** LSV curves of Pt/C and IrO_2_/C. 6

**Supporting Information, S6.** Raman scattering spectra of carbon alloys. 7

**Supporting Information, S7.** TGA curves of BMs, CNFs, and composites. 8

**Supporting Information, S8.** SEM images of pyrolyzed samples 9

**Supporting Information, S1.** Extraction protocol of CNFs from ascidian.

Ascidian tunicates were crushed by biaxial type shear crusher (Ujiie, Japan) and then, trace of seashell scaffolds was removed by washing with water. After coarse grinding at 18,500 rpm for 10 min with mechanical mixer (FMI, Japan), proteins were degraded by 1 % protease (Thermoase PC10F, Amano Enzyme, Japan) at 65 ˚C for 2 hrs. The proteins degraded material was treated a 22 % (w/w) solution of sodium hypochlorite pentahydrate (NaClO·5H_2_O) at 30 ˚C for 6 hrs. The dispersion was dispersed by fine grinding byusing mechanical homogenizer (Microtech Nichion, Japan) equipped with a 20 mm-diameter shaft at 7,500 rpm for 5 min and concentrated by centrifugation to prepare 2 wt % dispersion. The histogram of CNFs width (Figure S1) was measured by SEM images by using imaging software (ImageJ, NIH, USA). From the histogram, the maximum, the minimum, and average width of CNFs are 48.1 nm, 15.3 nm, and 28.9 nm, respectively.

**
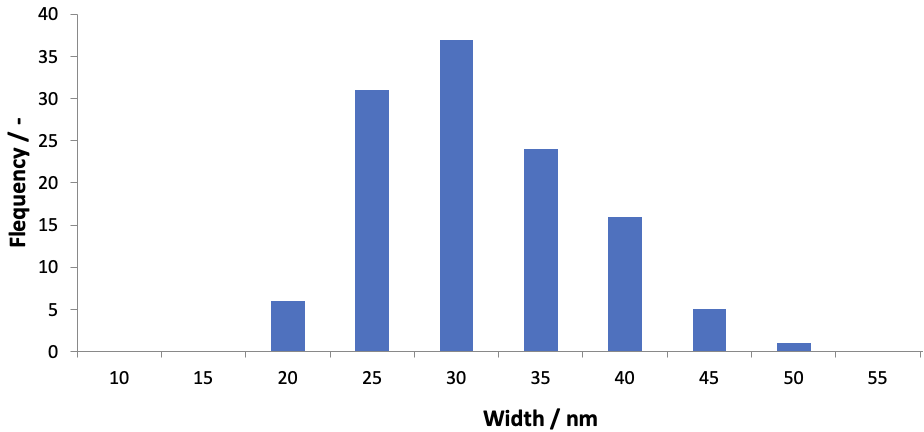
**

**Figure S1.** Histogram of CNFs width.

**Supporting Information, S2.** LSV curves of a carbon alloy before/after CN^-^ poisoning.


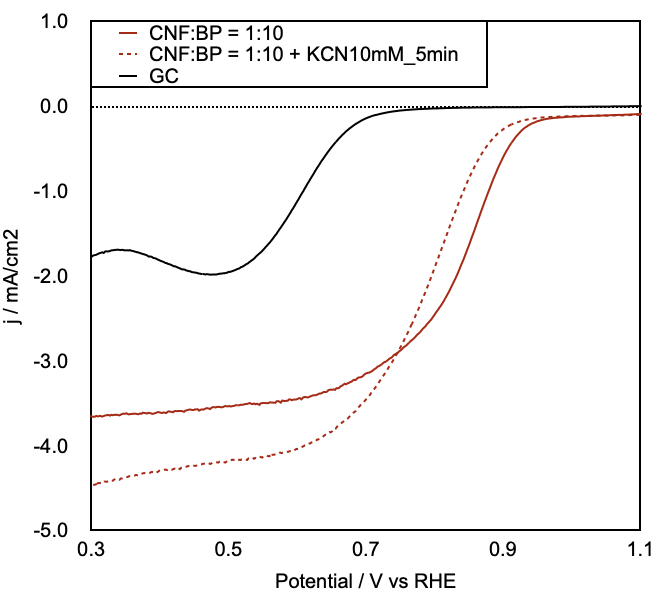


**Figure S2**. LSV curves of GC (Black line), CA-1/10-900 °C (red line), and CA-1/10-900 °C immersed in 10 mM KCN aq. for 5 min (red dashed line), respectively.

**Supporting Information, S3.** LSV curves of carbon alloys with different rotating speed.


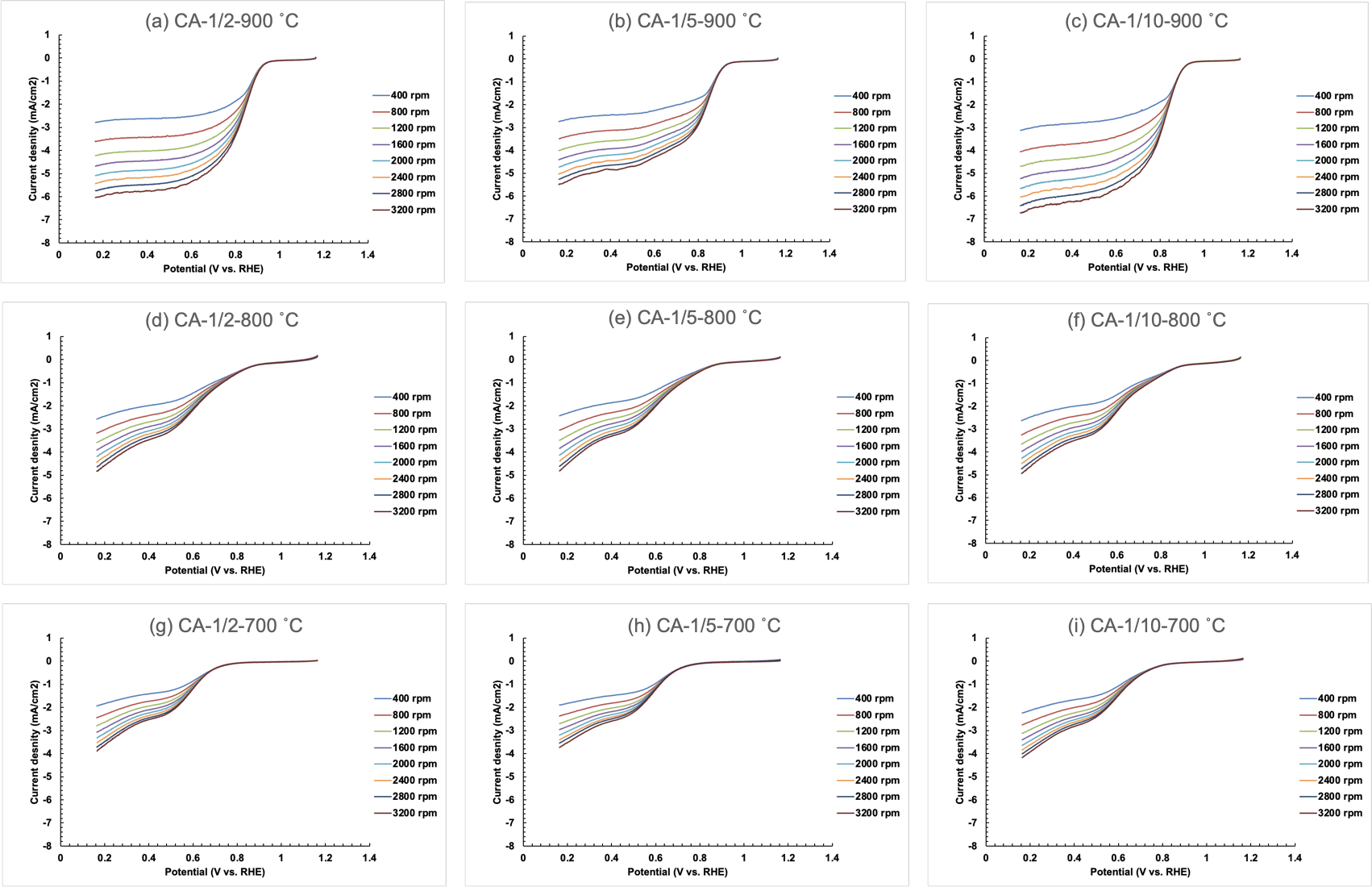


**Figure S3.** Plots of LSV curves of carbon alloys for ORR in 0.1 M KOH aq. (pH 13) with retaining speed ranging from 400 rpm to 3,200 rpm. (a) CA-1/2-900 ˚C, (b) CA-1/5-900 ˚C, (c) CA-1/10-900 ˚C, (d)CA-1/2-800 ˚C, (e) CA-1/5-800 ˚C, (f) CA-1/10-800 ˚C, (g)CA-1/2-700 ˚C, (h) CA-1/5-700 ˚C, (i) CA-1/10-700 ˚C, respectively.

**Supporting Information, S4.** SEM images and LSV curves of pyrolyzed BMs and CNFs.

Figure S4 shows LSV curves of GC, BMs and CNFs before (a), (b) and after (c), (d) pyrolyzed at 900 ˚C. Figure S5 shows LSV curves of GC, BMs, CNFs, and CA-1/10-900 ˚C measured by RRDE as same as other carbon alloy samples in order to evaluate ORR performances. The measurements were performed in the 0.1 M KOH aq. (pH 13) saturated with O_2_. On Comparison with the GC electrode, the BM-900 ˚C and CNF-900 ˚C had higher ORR performance, but the performance is relatively lower than that of CA-1/10-900 ˚C. It is noteworthy that the performance of the alloyed materials was the highest while that of pyrolyzed original materials was much lower. This result indicates the alloying process is required to improve ORR performance.


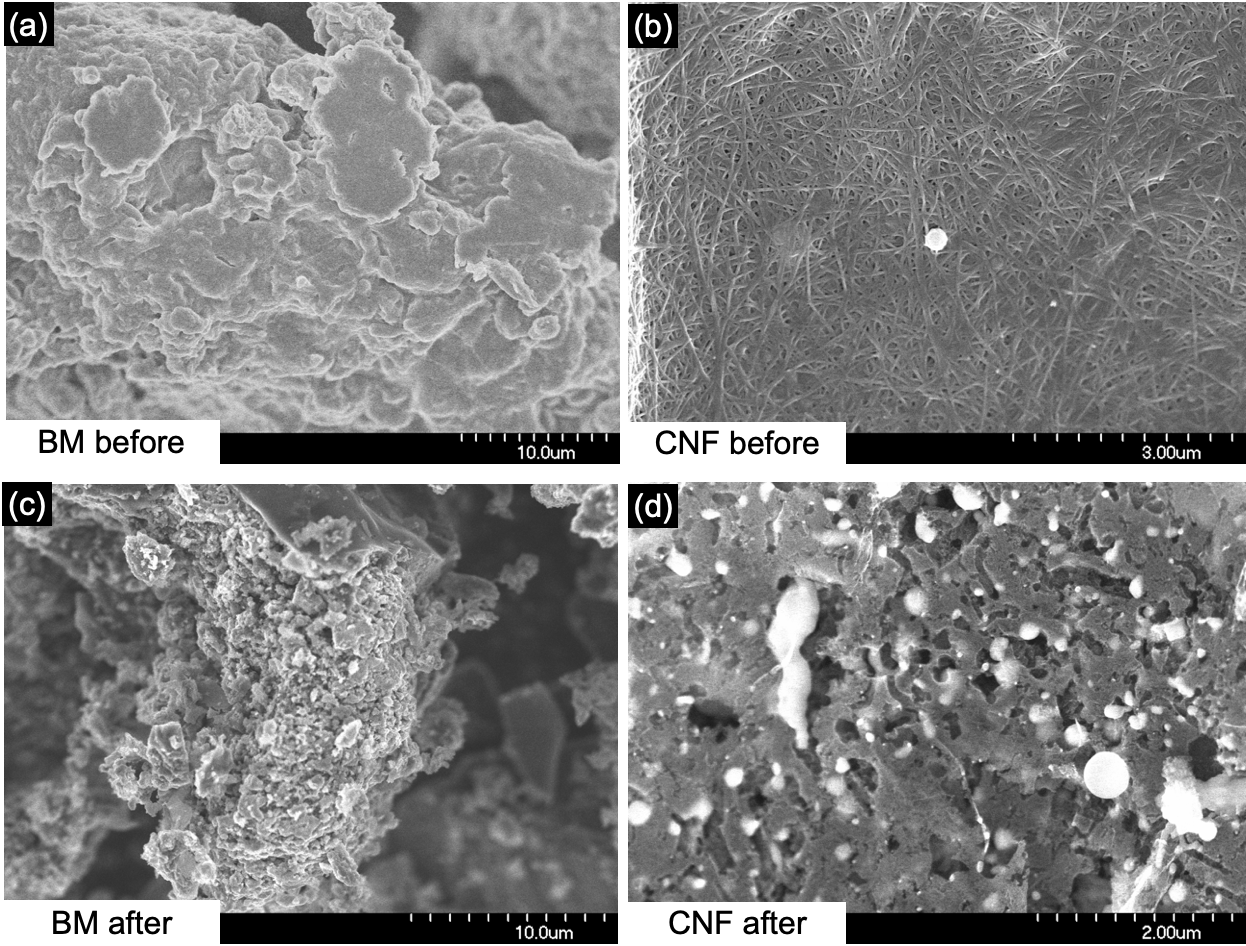


**Figure S4.** SEM images of BMs and CNFs before [(a), (b)] and after [(c), (d)] pyrolysis at 900 ˚C.


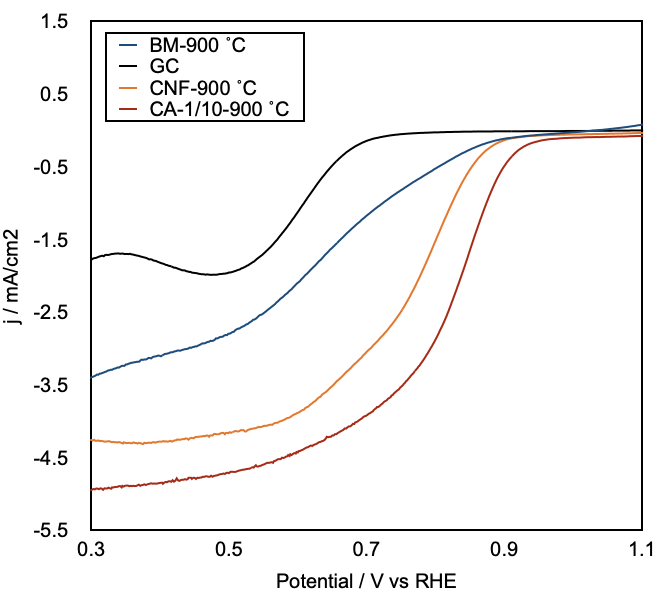


**Figure S5.** LSV curves of GC (black solid line), BMs (blue solid line) and CNFs (orange solid line) pyrolyzed at 900 ˚C, and CA-1/10-900 ˚C (red solid line), respectively.

**Supporting Information, S5.** LSV curves of Pt/C and IrO_2_/C.

LSV curves of Pt/C and IrO_2_/C for ORR/OER were measured under same conditions as carbon alloy samples. Figure S6 shows LSV curves of Pt/C, IrO_2_/C, CA-1/100-900 ˚C, and GC in the ORR region. Much higher *E*_onset(ORR)_ values of Pt/C, IrO_2_/C and CA-1/100-900 ˚C than that of GC were clearly observed. The observed trend in *E*_onset(ORR)_ was as follows: Pt/C> CA-1/100-900 ˚C > IrO_2_/C >> GC. Additionally, LSV curves of OER region were also measured for all samples (Figure S7). *E*_onset(OER)_ trend observed was IrO_2_/C~ CA-1/100-900 ˚C> Pt/C>> GC.


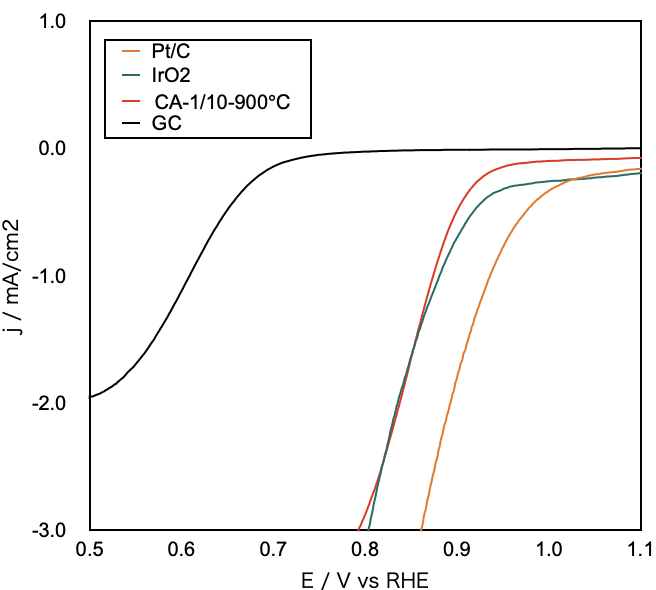


**Figure S6**. LSV curves of samples in the ORR region measured in O_2_-saturated 0.1 M KOH aq.


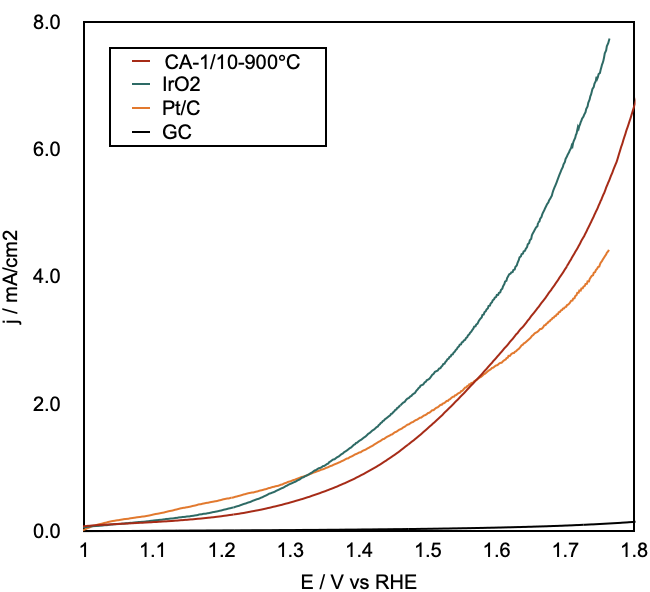


**Figure S7**. LSV curves of samples in the OER region measured in O_2_-saturated 0.1 M KOH aq.

**Supporting Information, S6.** Raman scattering spectra of carbon alloys.

Raman scattering of samples were measured by using Raman microscope (inVia Reflex, Renishaw, Gloucestershire, UK). Carbon alloys were fixed on carbon tapes and illuminated 532 nm incident light through a 20X objective lens whose intensity was 10% of maximum with using a grating of 1,800 lines. The signals were integrated 10 times.

From the Raman scattering spectra, broad peaks attributed to defect (D) and graphite (G) structures were found at 1,350 cm^-1^ and 1580 cm^-1^, respectively. The ratio between those two peak intensities represents quality of the carbon materials. From the Raman spectra of CA-1/10-700, 800, and 900 ˚C, the D/G ratio is almost constant in each case, and the ratio was c. a. 1, which means the quality of the carbon obtained from CNFs and BMs were close to commercially available carbon blacks. Also, the defect band gradually increased with increasing the pyrolysis temperature. These results indicated that amounts of hetero-atom doping, which form defects in the graphite networks.


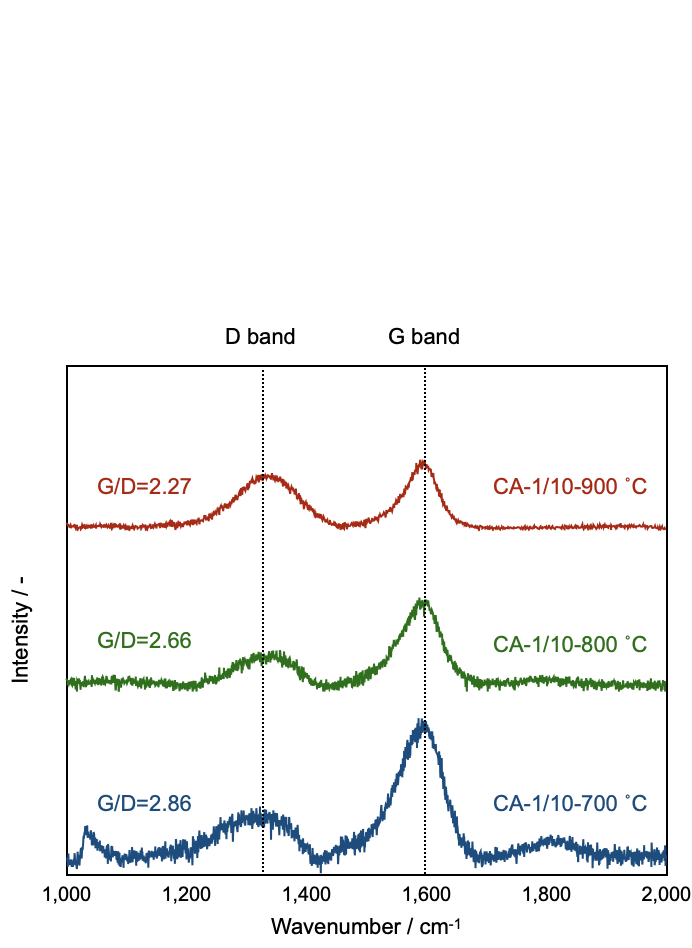


**Figure S8**. Raman spectra of CA-1/10-700 (blue), 800 (green), and 900 ˚C (red), respectively.

**Supporting Information, S7.** TGA curves of BMs, CNFs, and composites.

**Figure S9.** TGA curves of BMs (a), CNFs (b) and composites (c), respectively. The data indicates pyrolyzed BMs are mainly remained at higher temperature even though small amount of CNFs were remained.

**Supporting Information, S8.** SEM images of pyrolyzed samples


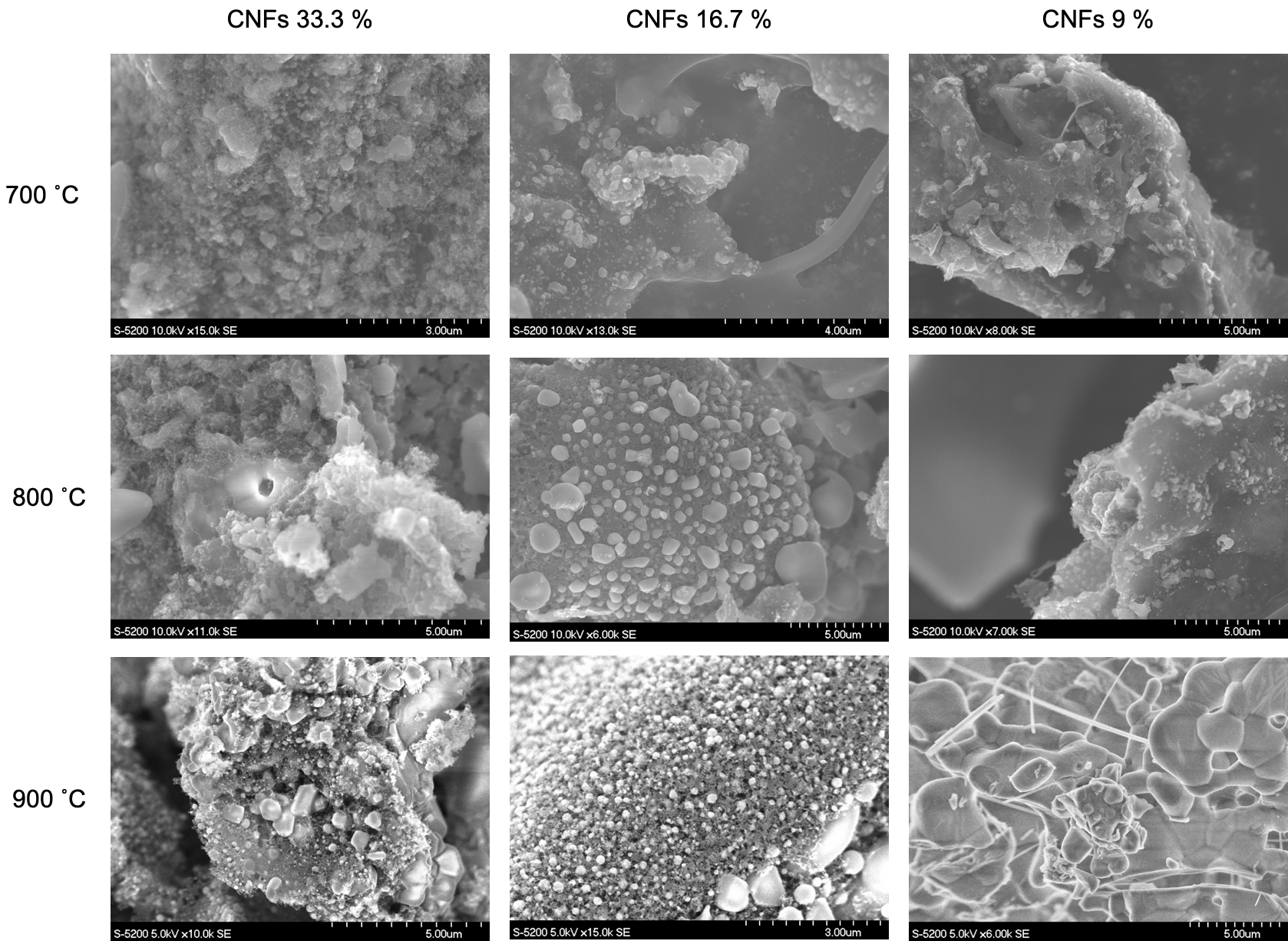


**Figure S10.** SEM images of pyrolyzed carbon alloys with different CNF contents at different pyrolyzed temperatures.
